# Fe(III) mineral reduction followed by partial dissolution and reactive oxygen species generation during 2,4,6-trinitrotoluene transformation by the aerobic yeast *Yarrowia lipolytica*

**DOI:** 10.1186/s13568-014-0094-z

**Published:** 2015-02-01

**Authors:** Ayrat M Ziganshin, Elvira E Ziganshina, James Byrne, Robin Gerlach, Ellen Struve, Timur Biktagirov, Alexander Rodionov, Andreas Kappler

**Affiliations:** Department of Microbiology, Kazan (Volga Region) Federal University, ul. Kremlyovskaya 18, Kazan, 420008 The Republic of Tatarstan Russia; Geomicrobiology, Center for Applied Geosciences, University of Tübingen, Tübingen, D-72076 Germany; Department of Chemical and Biological Engineering, Center for Biofilm Engineering, Montana State University, Bozeman, Montana 59717 USA; Institute of Physics, Kazan (Volga Region) Federal University, Kazan, 420008 The Republic of Tatarstan Russia

**Keywords:** 2,4,6-Trinitrotoluene, Biodegradation, Ferric (oxyhydr)oxides, *Yarrowia lipolytica*, Reactive oxygen species, Reactive nitrogen species

## Abstract

Understanding the factors that influence pollutant transformation in the presence of ferric (oxyhydr)oxides is crucial to the efficient application of different remediation strategies. In this study we determined the effect of goethite, hematite, magnetite and ferrihydrite on the transformation of 2,4,6-trinitrotoluene (TNT) by *Yarrowia lipolytica* AN-L15. The presence of ferric (oxyhydr)oxides led to a small decrease in the rate of TNT removal. In all cases, a significant release of NO_2_^−^ from TNT and further NO_2_^−^ oxidation to NO_3_^−^ was observed. A fraction of the released NO_2_^−^ was abiotically decomposed to NO and NO_2_, and then NO was likely oxidized abiotically to NO_2_ by O_2_. ESR analysis revealed the generation of superoxide in the culture medium; its further protonation at low pH resulted in the formation of hydroperoxyl radical. Presumably, a fraction of NO released during TNT degradation reacted with superoxide and formed peroxynitrite, which was further rearranged to NO_3_^−^ at the acidic pH values observed in this study. A transformation and reduction of ferric (oxyhydr)oxides followed by partial dissolution (in the range of 7–86% of the initial Fe(III)) were observed in the presence of cells and TNT. Mössbauer spectroscopy showed some minor changes for goethite, magnetite and ferrihydrite samples during their incubation with *Y. lipolytica* and TNT. This study shows that i) reactive oxygen and nitrogen species generated during TNT transformation by *Y. lipolytica* participate in the abiotic conversion of TNT and ii) the presence of iron(III) minerals leads to a minor decrease in TNT transformation.

**Electronic supplementary material:**

The online version of this article (doi:10.1186/s13568-014-0094-z) contains supplementary material, which is available to authorized users.

## Introduction

Nitroaromatic compounds have multiple applications in the chemical industry, amongst which special attention should be paid to 2,4,6-trinitrotoluene (TNT) due to its extensive use throughout the world (Harter 1985). A significant number of land and water territories are contaminated with this explosive and its conversion products as a result of the manufacture, transportation, storage and testing of munitions. These compounds are classified as relatively recalcitrant to biological degradation and their fate is affected by biogeochemical processes (Smets et al. 2007; Singh et al. 2012; Ziganshin and Gerlach 2014; Chien et al. 2014).

On the one hand, bioremediation strategies could offer attractive solutions for soil and water decontamination, although, TNT is exceedingly recalcitrant to complete biological mineralization. On the other hand, TNT nitro groups can be fairly easily reduced to hydroxylamino and amino derivatives by bacteria and fungi. However, these metabolites are also known to be toxic, potentially mutagenic and persistent contaminants (Smets et al. 2007; Singh et al. 2012; Khan et al. 2013). The most promising and efficient pathway of nitrogen release from TNT is the transformation of parent compound through the addition of hydride ions resulting in the formation of Meisenheimer hydride complexes followed by their subsequent destruction (ring fission) (Pak et al. 2000; Jain et al. 2004; Wittich et al. 2009; Ziganshin et al. 2010a). Therefore, the application of microorganisms, which are able to degrade TNT and its metabolites, can be useful for bioremediation of TNT-contaminated soil and water.

Iron is one of the most abundant elements in the biosphere, naturally occurring in the form of various Fe(II)- and Fe(III)-bearing minerals and present at many TNT-contaminated field sites. The biogeochemical cycling of iron also has an influence on nitroaromatic compounds, including TNT, mostly through the stimulation of nitro group reduction process in different soils, sediments and waters. In almost all cases TNT transformation in the presence of iron species is accompanied by the enhanced formation and accumulation of undesirable metabolites, such as hydroxylamino-dinitrotoluenes (HADNTs), amino-dinitrotoluenes (ADNTs) and/or diamino-nitrotoluenes (DANTs) (Hofstetter et al. 1999; Borch et al. 2005; Oh et al. 2008; Boparai et al. 2010), which are fairly persistent and approximately as toxic as TNT itself (Smets et al. 2007; Khan et al. 2013). Only a few studies have reported denitration of TNT in the presence of ferrihydrite (Eyers et al. 2008; Khilyas et al. 2013). In terms of the efficiency of remediation strategies, TNT denitration processes resulting in nitro group elimination would be advantageous if the nitro group reduction pathways could be minimized. Therefore, for the development of effective TNT-bioremediation technologies in the presence of iron-containing minerals and because of the distinct solubilities and reactivities of various Fe(III) minerals (Borch et al. 2010; Posth et al. 2014), it is important to investigate TNT transformation processes in the presence of different ferric (oxyhydr)oxides. In addition, very little is known about the reduction of Fe(III) minerals by strictly aerobic bacteria and yeasts in the presence of polynitroaromatic compounds.

Systematic data showing the transformation of TNT by distinct microorganisms in the presence of iron (oxyhydr)oxides under oxic conditions is lacking, despite the fact that oxygen can have a significant effect on Fe-transformation processes (Morgan and Lahav 2007). Only a few studies have so far demonstrated the generation of the superoxide radical anion (O_2_^·–^) during TNT reduction by purified enzymes (Kumagai et al. 2000; Kumagai et al. 2004) and in the presence of bacterial cells (Naumenko et al. 2008) via the formation of TNT nitroanion radicals. In some studies the involvement of abiotically and biologically produced O_2_^·–^ in the denitration of TNT and its metabolites was also reported (Fritsche et al. 2000; Van Aken and Agathos 2002; Stenuit et al. 2012). However, little is known regarding the biological production of O_2_^·–^ and other reactive oxygen species in culture medium during the microbial transformation of polynitroaromatic xenobiotics.

Given the importance of iron minerals for the fate of TNT, the main goal of this study was to investigate the effect of different ferric (oxyhydr)oxides on the TNT transformation processes by the yeast strain *Yarrowia lipolytica* AN-L15 and to identify iron mineral transformation in the presence of yeast cells and mineral additions of goethite (α-FeOOH), hematite (α-Fe_2_O_3_), magnetite (Fe_3_O_4_) or ferrihydrite (5Fe_2_O_3_^●^9H_2_O). Finally, reactive oxygen and nitrogen species generated over the course of TNT transformation were characterized.

## Materials and methods

### Yeast growth media and growth conditions

The yeast strain *Yarrowia lipolytica* AN-L15 deposited in the Russian National Collection of Industrial Microorganisms under a collection number of VKPM Y-3492 was cultivated aerobically at 30°C for 24 hours in Petri dishes with Sabouraud agar medium containing (per liter) 10 g of glucose, 10 g of peptone, 5 g of yeast extract, 0.25 g of NaCl and 20 g of agar (Ziganshin et al. 2007a). Yeast cells were harvested, washed twice with 16 mM phosphate buffer (pH 6.0) and added into 250 mL Erlenmeyer flasks containing 50 mL of a synthetic medium of the following content (mM): glucose, 28; (NH_4_)_2_SO_4_, 7.6; MgSO_4_, 2; Na_2_HPO_4_, 1.94; KH_2_PO_4_, 14.06 (pH 6.0). In ferric (oxyhydr)oxide containing treatments, goethite, hematite, magnetite or ferrihydrite were added to the synthetic medium to a final concentration of 0.15 g L^−1^ or 0.3 g L^−1^ (measured as Fe). As ferric (oxyhydr)oxides, commercially available goethite, hematite and magnetite were used (Lanxess). In the case of ferrihydrite-containing experiments, 2-line ferrihydrite was synthesized by dissolving of 4.0 g of Fe(NO_3_)_3_^●^9H_2_O in 50 mL of Millipore water following Amstaetter et al. (2012). 1 M KOH was then added dropwise over 3 min until a pH of 7.3 was reached. After 2 h, the pH was adjusted to a final value of 7.5. After centrifugation at 5,000 × g for 10 min and washing of the solid phase with Millipore water, 2-line ferrihydrite was obtained and used for the experiments. Cell growth was monitored photometrically by measuring the optical density at 600 nm (OD_600_) using a SPECOL 1300 spectrophotometer (Analytik Jena, Germany) with cell-free supernatant as a reference; the initial OD_600_ was adjusted to 0.05. TNT was added to a final concentration of 0.44 mM from an ethanolic stock solution (0.8 ml of 96% ethanol into 50 ml of medium), and the culture was incubated in the absence of light at 30°C on a rotary shaker at 150 rpm. Experiments conducted in the absence of TNT contained the same amount of 96% ethanol. All experiments were set up in triplicate.

### Analytical methods

TNT and biotransformation products were detected and quantified with a Shimadzu high-performance liquid chromatograph equipped with an autoinjector, a diode array detector, a column oven, a Supelcosil LC-8 guard column and a Supelcosil octyl (C-8) column (150 by 4.6 mm; particle size, 5 μm) as described previously (Ziganshin et al. 2007b; Ziganshin et al. 2010b).

The nitrite and nitrate ions in the culture fluid were determined by using an AA3 SEAL Analyzer equipped with a XY2 Sampler (SEAL Analytical, Germany).

Dissolved Fe(II) concentrations were quantified after sample centrifugation at 15,000 × g for 10 min and subsequent filtration through 0.2 μm filters (Spartan 13/0.2 RC; Whatman). Filtered sample (100 μL) was added to 900 μL of 1 M HCl. The received solution (20 μL) was added to 80 μL of 1 M HCl and to 100 μL of 0.1% ferrozine solution in 96-well microtiter plates and incubated for 5 min at room temperature. The resulting absorbance (562 nm) in the plates was measured using a Flashscan 550 plate reader (Analytik Jena, Germany) (Stookey 1970).

For ^57^Fe Mössbauer spectroscopy, biologically produced mineral precipitates were centrifuged at 10,000 × g for 10 min and then dried in an anoxic glovebox (100% N_2_). Samples were prepared by loading dried powders into Plexiglas holders (area 1 cm^2^). In order to ensure a homogeneous sample with ideal thickness, each sample was mixed and ground using a pestle and mortar with 80 mg glucose monohydrate. The samples were transferred to the Mössbauer spectrograph and inserted into a closed-cycle exchange gas cryostat (Janis cryogenics). Spectra were collected at 295 K, 77 K and 5 K by using a constant acceleration drive system (WissEL) in transmission mode with a ^57^Co/Rh source and calibrated against a 7 μm thick α-^57^Fe foil measured at room temperature. Spectra were analyzed using Recoil (University of Ottawa) using the Voigt Based Fitting (VBF) routine. The HWHM was fixed to 0.122 mm/s, as determined from the minimum line width of the calibration foil, measured at 295 K.

Superoxide anion radical (O_2_^·–^) generation during TNT transformation was determined by electron spin resonance (ESR) spectroscopy through the formation of 5-(Diethoxyphosphoryl)-5-methyl-1-pyrroline-N-oxide (DEPMPO) spin adducts (Frejaville et al. 1995). Firstly DEPMPO (50 mM) was added to a solution to be analyzed and after 30 sec incubation process at room temperature the mixture was transferred to glass tubes with an inner diameter of 1 mm (Sigma-Aldrich, Germany). ESR spectra were then recorded at room temperature with an ESP 300 Electron Spin Resonance spectrometer (Bruker, Germany) and a super-high Q microwave cavity. The experimental parameters were as follows: microwave power 2 mW, microwave frequency 9.73 GHz, modulation amplitude 0.2 mT, time constant 82 ms and scan rate 2.1 G/s. The received ESR spectra were analyzed using EasySpin toolbox for MATLAB. TNT-free or cell-free experiments were used as a control. The xanthine-xanthine oxidase reaction was used as the standard system for O_2_^·–^ generation; superoxide dismutase was used to remove superoxide anions in the samples (Stenuit et al. 2012).

### Chemicals

2,4,6-Trinitrotoluene and 2,4-dinitrotoluene (2,4-DNT) are the chemicals of ChemService (West Chester, USA); 2-amino-4,6-dinitrotoluene (2-ADNT) and 4-amino-2,6-dinitrotoluene (4-ADNT) were received from Supelco (Bellefonte, USA); 2-hydroxylamino-4,6-dinitrotoluene (2-HADNT) and 4-hydroxylamino-2,6-dinitrotoluene (4-HADNT) as well as some other potential metabolites of TNT nitro group reduction pathway were purchased from AccuStandard (New Haven, USA). DEPMPO as a spin trapping reagent for reactive oxygen species was received from Enzo Life Sciences (Farmingdale, USA). Hypoxanthine, xanthine oxidase, superoxide dismutase as well as all other chemicals and reagents were purchased from Sigma-Aldrich (Germany).

## Results

### Influence of ferric (oxyhydr)oxides on TNT biotransformation processes

**Figure 1:**
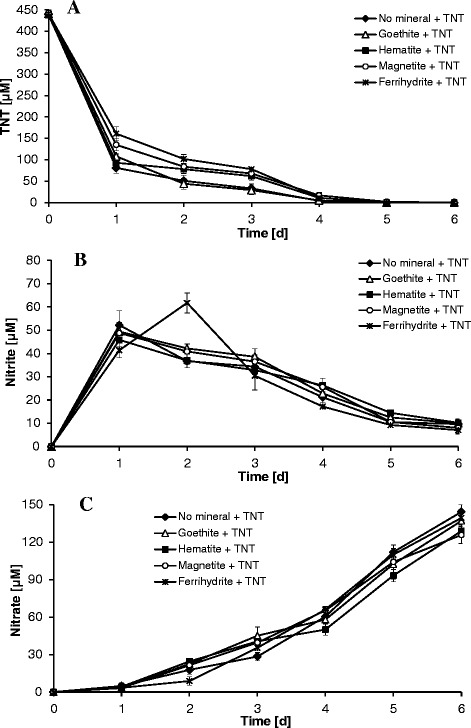
**TNT removal (А), nitrite accumulation and removal (B) and nitrate accumulation during transformation of TNT (C) by yeast cells of** ***Y. lipolytica*** **AN-L15 in the absence and presence of various ferric (oxyhydr)oxides.** Error bars represent the standard deviation of triplicate experiments.

The effect of various ferric (oxyhydr)oxides with a final concentration of 0.3 g L^−1^ (measured as Fe) on TNT (440 μM) transformation and denitration by *Yarrowia lipolytica* AN-L15 in the presence of glucose and ethanol as carbon and electron sources was evaluated. Independent of the presence of ferric (oxyhydr)oxides, yeast growth in the presence of TNT occurred with a pH decrease of the synthetic medium from an initial pH of 6.0 to below 3.0 because of production of organic acids (Additional file 1: Table S1, Supporting Information, SI). In the absence of TNT, the pH change of the medium was observed to decrease to almost the same values (data not shown). HPLC analysis demonstrated that TNT was efficiently removed from the medium during yeast growth (Figure 1A). As shown in Figure 1A, complete TNT removal by *Y. lipolytica* AN-L15 occurred within 5 days in the presence or absence of the various ferric (oxyhydr)oxides. The highest rate of TNT biotransformation was observed in the absence of iron minerals followed by the treatments in the presence of goethite. The presence of ferrihydrite, magnetite and hematite led to a small decrease in the rate of TNT removal. *Y. lipolytica* removed TNT in the range of 63–82% of TNT during the first day of incubation, while the remaining 18–37% were further transformed in the following 3 to 4 days of the experiments. The TNT biotransformation by *Y. lipolytica* was slower compared to previous studies (Ziganshin et al. 2010b; Khilyas et al. 2013), which can be explained by the lower initial yeast cell concentration used for these experiments (initial OD_600_ of 0.05). When an initial OD_600_ of 0.2 or 1.0 was used in Ziganshin et al. (2010b) and Khilyas et al. (2013), TNT was completely removed in the absence of ferric (oxyhydr)oxides within 24 and 10 hours, respectively.

During the conversion of TNT by yeast cells of *Y. lipolytica* AN-L15, different metabolites successively appeared in the culture medium. Among the aromatic ring hydrogenation products, eight hydride-Meisenheimer complexes of TNT were detected, which were previously described in detail (Ziganshin et al. 2007b; Ziganshin et al. 2010b). Independent of the presence of ferric (oxyhydr)oxides, the main intermediate metabolite detected during the first day of incubation was a monohydride-Meisenheimer complex of TNT (3-H^−^-TNT). The maximum concentration of 3-H^−^-TNT detected after 1 day ranged from 255–294 μM (data not shown); its concentration was estimated as described previously (Ziganshin et al. 2007b). Its subsequent biotransformation into 2,4-DNT and other dihydride-Meisenheimer complexes of TNT (3,5-2H^−^-TNT and 3 isomers of 3,5-2H^−^-TNT · H^+^) as well as degradation of TNT-dihydride complexes over the next 3–4 days occurred with the liberation of nitrite into the medium in the absence as well as in the presence of the different ferric (oxyhydr)oxides (Figure 1B). As can be seen in Figures 1A and 1B, the maximum amount of nitrite detected during TNT removal was in the range of 45–61 μM. In the presence of ferrihydrite, TNT transformation was slightly less significant combined with delayed nitrite ion liberation, which might be due to the increase in buffer capacity of the medium in the presence of ferrihydrite as asserted by Khilyas et al. (2013). After 1 day, nitrate was also detected indicating nitrite oxidation to nitrate (Figure 1C). The highest concentration of nitrate was observed in iron mineral-free systems (145 ± 7 μM), whereas the lowest nitrate accumulation was found in the culture medium containing magnetite (126 ± 7 μM) and hematite (129 ± 5 μM). Interestingly, 2,4-DNT as a product of TNT degradation was found at high levels only in iron mineral-free treatments (72 ± 7 μM), while the addition of ferric (oxyhydr)oxides resulted in lower concentrations of this metabolite (15–46 μM; SI, Additional file 1: Table S1). TNT nitro group reduction metabolites, such as HADNTs and ADNTs, were also detected and their concentrations are summarized in Additional file 1: Table S1 (SI). Additional abiotic experiments conducted in the presence of iron minerals at pH 6.0 or 7.0 showed that nitrite, nitrate as well as TNT were relatively stable and did not undergo any transformation reactions in the absence of yeast cells (data not shown). Also, we were unable to detect other potential conversion products of TNT such as diarylamines, tetranitro-azoxytoluenes, tetranitro-azotoluenes, amino-dimethyl-tetranitrobiphenyls or Bamberger rearrangement products during aerobic growth of *Y. lipolytica* AN-L15 in the presence of Fe(III) minerals.

### Superoxide generation during TNT biotransformation

**Figure 2:**
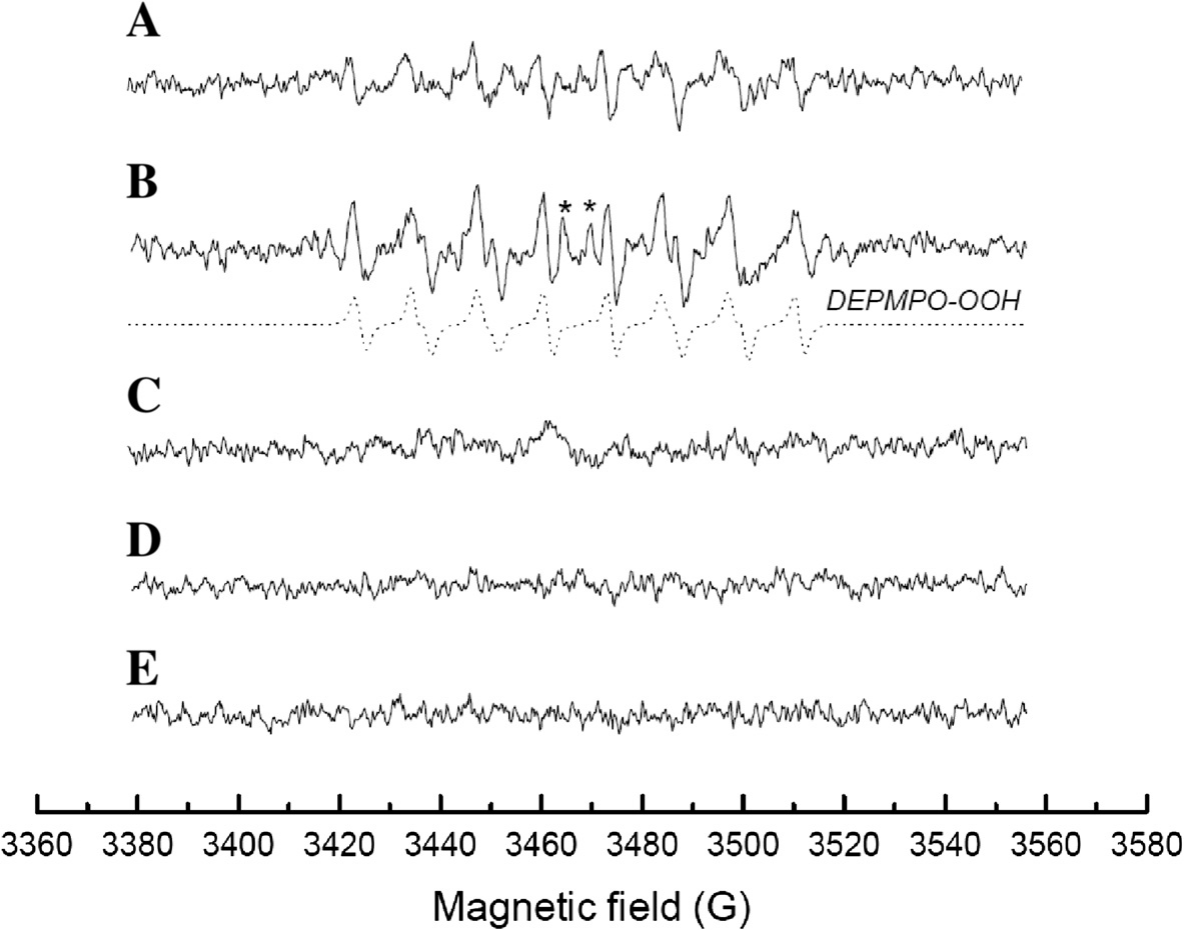
**ESR spectra of DEPMPO spin adducts generated in the presence of yeast cells of** ***Y. lipolytica*** **AN-L15 and TNT in the absence of Fe(III) (oxyhydr)oxides (measured 30 minutes after the start of the experiment) (A) and in a standard xanthine/xanthine oxidase system (reference spectrum for DEPMPO-OOH). (B)** Simulated spectrum of DEPMPO-OOH is presented as a reference (dashed line); the typical set of hyperfine coupling constants was used for simulation (Frejaville et al. 1995). The spectral lines attributed to DEPMPO-OH spin adduct are marked with asterisks; their appearance is the result of DEPMPO-OOH decomposition to DEPMPO-OH (Stenuit et al. 2012). **(C)** Same as in **(A)** with the exception that superoxide dismutase (200 U mL^−1^) was added. Control experiments in the presence of *Y. lipolytica* AN-L15 and absence of TNT **(D)** and in the absence of *Y. lipolytica* AN-L15 and presence of TNT **(E)**. The spectra were recorded for 10 min after the DEPMPO was added. The experimental parameters were as follows: microwave power 2 mW, microwave frequency 9.73 GHz, modulation amplitude 0.2 mT, time constant 82 ms and scan rate 2.1 G/s.

ESR spectra of DEPMPO radical adducts obtained during TNT biotransformation clearly showed the generation of O_2_^·–^ radical species in the culture medium (Figure 2A). This ESR spectrum was obtained 30 minutes after the start of the experiment when the culture medium pH was around 6.0. Superoxide anion presents in equilibrium with its protonated form, hydroperoxyl radical (HO_2_^·^), in an aqueous solution, and this equilibrium is shifted towards HO_2_^·^ under acidic conditions (Bielski et al. 1985). Therefore, a fraction of superoxide anions is expected to be protonated at the low pH values observed in the present work. Both species, O_2_^·–^ and HO_2_^·^, can react with DEPMPO yielding the same spin adduct (DEPMPO-OOH) (Lauricella et al. 2004). Therefore, the nitroxide radical associated with the superoxide/hydroperoxyl conjugate pair will be referred to as DEPMPO-superoxide spin adduct hereafter. For confirmation of the lines in the spectra obtained during the TNT biotransformation namely to DEPMPO-OOH, a standard xanthine/xanthine oxidase reaction was used that generates O_2_^·–^ (Figure 2B). The standard spectrum was compared with those received during TNT transformation by *Y. lipolytica* AN-L15 and associated with the generation of O_2_^·–^. The addition of superoxide dismutase (SOD) removed the original signal, indicating that this mechanism was superoxide-dependent and did not lead to hydroxyl radical generation (Figure 2C). Since SOD was able to remove the original signal, the appearance of spectral lines attributed to the hydroxyl radical spin adduct (DEPMPO-OH) is the result of DEPMPO-OOH decomposition to DEPMPO-OH (Stenuit et al. 2012). This suggests that TNT transformation in the presence of the yeast strain results in the formation of O_2_^·–^/HO_2_^·^ in the culture medium. However, we cannot exclude the partial generation of hydroxyl radicals (HO^.^) as well as other similarly reactive species in these systems. Control experiments in the presence of *Y. lipolytica* AN-L15 but absence of TNT, as well as those in the absence of *Y. lipolytica* AN-L15 and presence of TNT did not result in the generation of O_2_^·–^ (Figure 2D and 2E). Additional experiments conducted in the presence of yeast cells of *Y. lipolytica*, different ferric (oxyhydr)oxides and TNT led to formation of the same ESR spectra, indicating the formation of superoxide anions as well (results not shown).

### Reduction of ferric (oxyhydr)oxides during incubation with *Y. lipolytica* in the presence of TNT

**Figure 3:**
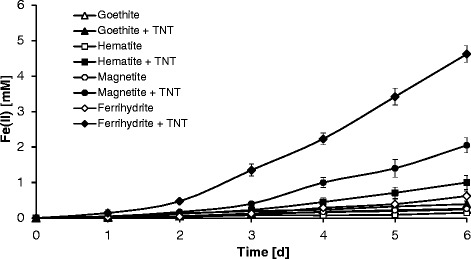
**Formation of dissolved Fe(II) during aerobic growth of** ***Y. lipolytica*** **AN-L15 in the presence of different ferric (oxyhydr)oxides (0.3 g L** ^**−1**^ **Fe).** Error bars represent the standard deviation of triplicate experiments.

Ferric (oxyhydr)oxides dissolution by aerobically growing *Y. lipolytica* AN-L15 cells was observed in the presence of TNT with the accumulation of dissolved Fe(II) (Figure 3). All ferric (oxyhydr)oxides (0.3 g L^−1^ Fe) tested in this research appeared to undergo partial reduction to Fe(II) in TNT-containing synthetic medium in the presence of *Y. lipolytica* AN-L15. The greatest extent of Fe(III) reduction was observed when ferrihydrite was used for the experiments, while some dissolved Fe(II) production was also observed in magnetite-containing treatments. Even less dissolved Fe(II) production was observed in the presence of hematite and goethite. After 6 days, the concentrations of dissolved Fe(II) in TNT- and yeast-containing medium in the presence of ferrihydrite, magnetite, hematite and goethite reached 4.62 mM, 2.05 mM, 1.02 mM and 0.39 mM, respectively (Figure 3). Approximately 86% of the Fe(III) added as ferrihydrite had been converted into Fe(II), while for the other ferric minerals, the extent of Fe(III) conversion to Fe(II) was less significant. Approximately 38, 19 and 7% of Fe(III) were liberated through dissolution when magnetite, hematite and goethite were used, respectively.

In contrast, significantly lower amounts of ferrous iron production were observed in TNT-free setups and in the presence of *Y. lipolytica* AN-L15 when compared to TNT-containing treatments. The maximum concentrations of dissolved Fe(II) in the absence of TNT after 6 days of incubation with strain AN-L15 were 0.62 mM, 0.26 mM, 0.2 mM and 0.2 mM (approximately 12, 5, 4 and 4% of the added ferrihydrite, magnetite, hematite and goethite, respectively; Figure 3). When lower concentrations of ferric (oxyhydr)oxides (0.15 g L^−1^ Fe) were tested, lower amounts of Fe(II) were detected (SI, Additional file 1: Figure S1); however, the trends remained the same. In control experiments, using cell-free medium, no Fe(II) accumulation was observed regardless of the presence of TNT (data not shown).

### Transformation of ferric (oxyhydr)oxides during incubation with *Y. lipolytica* in the absence/presence of TNT

**Figure 4:**
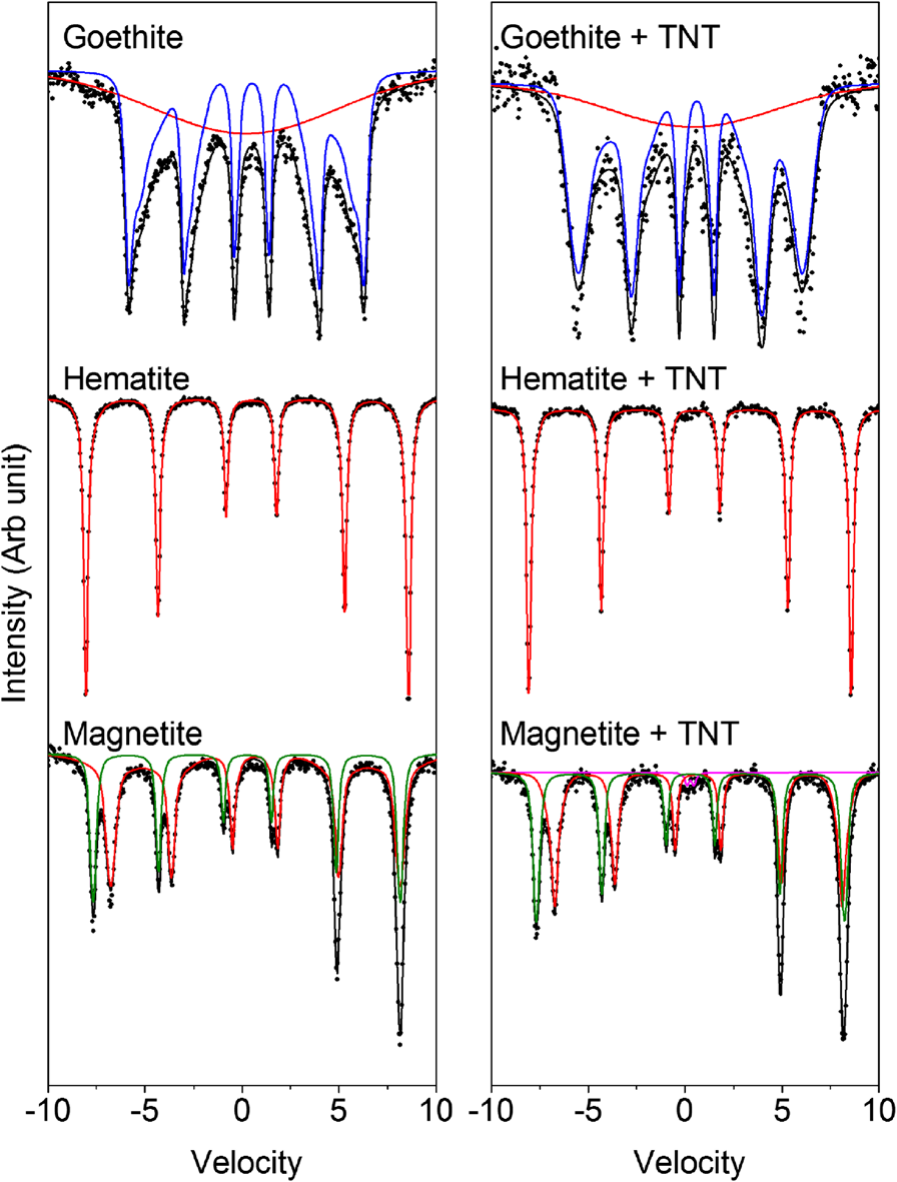
**Mössbauer spectra obtained for samples goethite, hematite and magnetite at room temperature (295 K) after 6 days of incubation in the presence of** ***Y. lipolytica*** **AN-L15 with and without TNT.**

**Figure 5:**
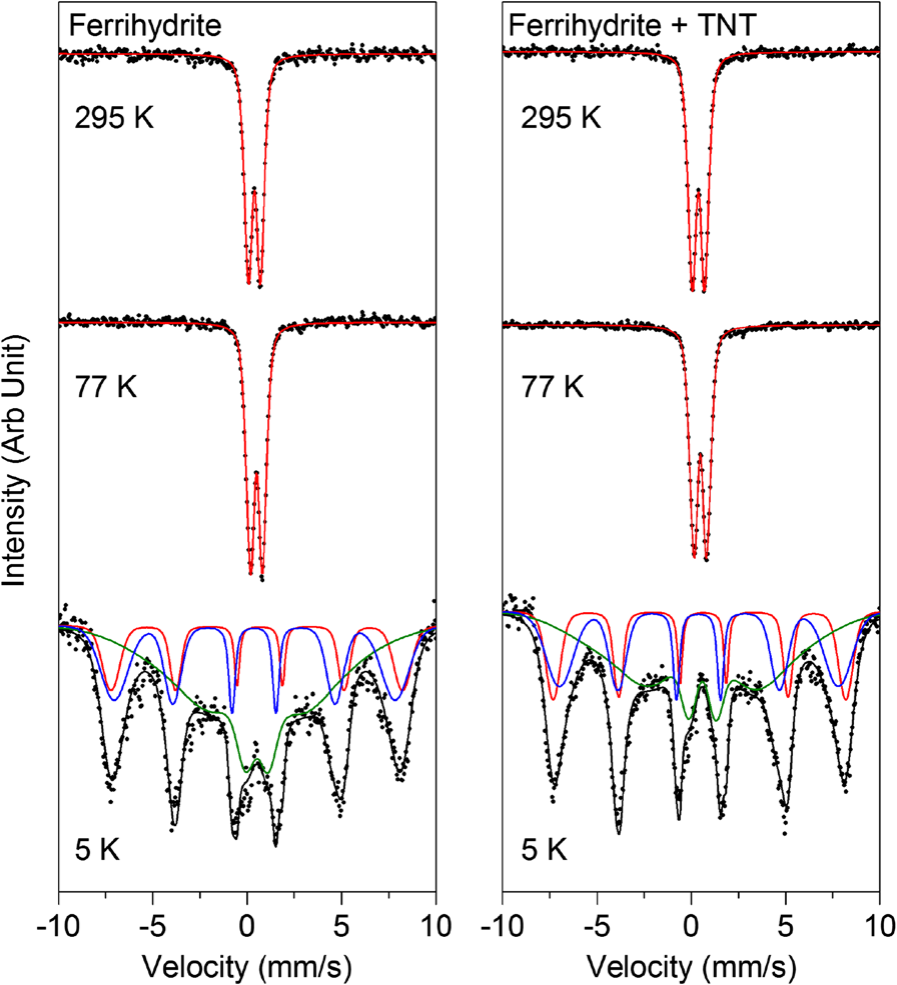
**Mössbauer spectra of ferrihydrite after 6 days of incubation in the presence of** ***Y. lipolytica*** **AN-L15 with and without TNT (spectra collected at 295 K, 77 K and 5 K).**

^57^Fe Mössbauer spectroscopy was performed on all samples to determine the iron mineral phases present and to identify potential differences between the materials after a 6 day incubation period in the presence of *Y. lipolytica* AN-L15 cells alone, and also in the presence of yeast cells with TNT (Figures 4 and 5). Room temperature spectra for magnetite, hematite and goethite are shown in Figure 4, whilst Additional file 1: Table S2 (SI) shows the parameters obtained through spectral fitting of the different ferric (oxyhydr)oxides after aerobic incubation with *Y. lipolytica* AN-L15 cells in the absence or the presence of TNT.

Goethite is usually expected to have a narrow hyperfine field at room temperature (38.0 T) and a small center shift (0.37 mm/s). The spectra obtained for both goethite samples have a narrower hyperfine field than expected (33.1–33.9 T); however, they also both exhibit superparamagnetic relaxation. This occurs close to the blocking temperature of a material and is likely the result of small average crystallite size or poor crystallinity of the sample. The paramagnetic background decreases in area relative to the sextet corresponding to the goethite from 39.5% to 24.8% for goethite and goethite + TNT, respectively (Figure 4). A small increase in center shift (CS) from 0.37 mm/s (goethite) to 0.43 mm/s (goethite + TNT) was also observed.

Both hematite samples (+/− TNT) possess a single sextet with very wide magnetic splitting (51.4 T) and low center shift (0.37–0.38 mm/s) that is characteristic of natural and synthetic hematite (Cornell and Schwertmann 2003). Its incubation in the presence of *Y. lipolytica* cells alone and with yeast cells and TNT did not yield any additional mineral phases or differences from the parent mineral (Figure 4).

Magnetite samples exhibit two overlapping sextets which are characteristic of the iron mineral. One sextet corresponds to Fe^3+^ in tetrahedral coordination, denoted the A-site, or ^Tet^Fe^3+^. The second sextet is observed above the Verwey temperature of magnetite (~120 K) and is formed due to electron hopping within the octahedral lattice and corresponds to both Fe^2+^ and Fe^3+^. This second sextet is commonly denoted the B site, or ^Oct^Fe^2.5+^. The results obtained here indicate that the spectrum of a magnetite + cells + TNT sample can only be fully fitted with an additional doublet which contributes to 1.3% of the total area (Figure 4). This doublet has a very low CS of 0.28 mm/s and low quadrupole splitting (ΔE_Q_) of 0.35 mm/s, which is consistent with a poorly crystalline Fe(III) mineral phase; however, it is not clear what the mineral identity is.

Finally, Mössbauer spectra were collected for ferrihydrite samples at three different temperatures, 295 K, 77 K and 5 K (Figure 5). Both spectra collected at 295 K have the same center shift (0.38 mm/s) and very similar ΔE_Q_ (0.68 and 0.70 mm/s for samples of ferrihydrite incubated with *Y. lipolytica* AN-L15 and with *Y. lipolytica* AN-L15 and TNT, accordingly). These parameters are characteristic for ferrihydrite at room temperature (Cornell and Schwertmann 2003; Mikutta et al. 2008). Spectra obtained at 77 K maintain the paramagnetic doublet observed at room temperature, albeit with an increase in CS to 0.49 mm/s for both samples (Figure 5). Additional analyses were carried out at 5 K, with both samples requiring the fitting of two magnetically ordered (S1 and S2) and one poorly ordered (S3) sextets (Figure 5, Additional file 1: Table S2). The hyperfine fields of the ordered sextets are at close to the expected values of ferrihydrite (Eusterhues et al. 2008), although it is clear that neither sample is fully magnetically ordered, as seen by the requirement of a poorly ordered sextet. The total contribution of this background phase decreases from 55.0% (ferrihydrite + cells) to 49.9% (ferrihydrite + cells + TNT). Pure 2-line or 6-line ferrihydrite is usually expected to be fully magnetically split at 5 K and does not require this background feature to fit the data. This suggests that both samples have a very low blocking temperature, which is potentially much lower than the value expected for typical ferrihydrite (Murad 1996).

## Discussion

In this study we investigated the impact of various ferric (oxyhydr)oxides on TNT transformation by *Y. lipolytica* AN-L15. The fastest rate of TNT removal was seen in the absence of iron minerals followed by experiments in the presence of goethite. The presence of ferrihydrite, magnetite and hematite led to a small decrease in the rate of TNT transformation. However, significant TNT denitration by *Y. lipolytica* occurred in the absence and presence of ferric (oxyhydr)oxides, indicating the possibility of TNT aromatic ring cleavage independent of the presence of iron minerals. The significant liberation of NO_2_^−^ from TNT and further NO_2_^−^ oxidation to NO_3_^−^ was observed with yeast cells of *Y. lipolytica*. Also, the presence of ferric (oxyhydr)oxides did not stimulate the nitro group reduction. It has previously been suggested that the increase of nitro group reduction metabolites during TNT bioconversion is possible in the presence of iron minerals without any denitration processes (Hofstetter et al. 1999; Borch et al. 2005). However, our results indicate that TNT denitration by aerobically grown *Y. lipolytica* AN-L15 can take place in iron rich environments, and that the presence of ferric (oxyhydr)oxides does not lead to an increase of HADNT production. Given that Fe(II)- and Fe(III)-containing minerals are found at many TNT-contaminated field sites, these results are of significance for TNT bioremediation in such environments.

ESR spectra of DEPMPO radical adducts obtained during TNT biotransformation clearly demonstrated the generation of O_2_^·–^/HO_2_^·^ radical species in the culture medium. Presumably, the generation of reactive oxygen species here is the result of the interaction of *Y. lipolytica* AN-L15 cells with TNT and its transformation products, resulting in TNT-induced oxidative stress. It is known that TNT can produce superoxide radical anions through TNT nitroanion radical formation (Kumagai et al. 2000; Kumagai et al. 2004; Naumenko et al. 2008). Recently, an involvement of abiotically produced superoxide radical anions in the TNT denitration process with the accumulation of nitrite ions in the reaction mixture was reported (Stenuit et al. 2012). The authors suggested that denitration occurred through a nucleophilic attack on the parent trinitroaromatic compound; however, the exact mechanism is yet unknown. The generation of superoxide anions in our treatments also indicates their possible participation in the TNT denitration process. However, the main pathway of nitrite/nitrate liberation from TNT by *Y. lipolytica* AN-L15 occurred via biodegradation of TNT-hydride complexes resulting in the formation of 2,4-DNT and other unidentified, carbon-containing metabolites.

In recent work we detected nitric oxide (NO) as a metabolite of TNT biodegradation by *Y. lipolytica* AN-L15 (Khilyas et al. 2013). A potential pathway for its formation is the abiotic conversion of nitrous acid (formed through nitrite release during Meisenheimer hydride complexes destruction) to NO and nitric acid (nitrate) via a disproportionation reaction at low pH (Van Cleemput and Baert 1984). It is also likely that a fraction of the released NO_2_^−^ was decomposed to NO and nitrogen dioxide (NO_2_) and the produced NO could have been oxidized to NO_2_ in the presence of molecular oxygen (O_2_) as ascertained by Klueglein and Kappler (2013). In addition, the produced NO_2_ may potentially be scavenged by natural organic matter (NOM) through nitration reactions. Another fraction of the released NO_2_^−^ could have reacted with amino group-containing cell components in the low pH systems with the formation of nitrosoamines (Fischer and Warneck 1996). All of these reactions might have lead to the decrease of the total amount of NO_2_^−^/NO_3_^−^ detected in our experimental systems.

Furthermore, NO released during TNT biodegradation by *Y. lipolytica* AN-L15 might have reacted with O_2_^·–^ to form peroxynitrite (ONOO^−^) which is a stronger oxidant than both NO and O_2_^·–^ (Pfeiffer et al. 1997; Vásquez-Vivar et al. 1997). Kumagai et al. (2004) demonstrated the production of NO and O_2_^·–^ in the presence of TNT catalyzed by nitric oxide synthase and also proposed a mechanism of peroxynitrite formation. Peroxynitrite is not stable under acidic conditions resulting in rearrangement to nitrate (Pryor and Squadrito 1995; Pfeiffer et al. 1997). Thus, together with the biological and abiotic oxidation of released NO_2_^−^ into NO_3_^−^, NO_3_^−^ accumulation during TNT biodegradation by *Y. lipolytica* AN-L15 at low pH could also occur through rearrangement of ONOO^−^. Some other oxidative reactions are also possible as ONOO^−^ reacts rapidly with CO_2_, producing highly oxidizing species such as NO_2_ and CO_3_^·–^ (Denicola et al. 1996). Since *Y. lipolytica* AN-L15 is able to synthesize and excrete different organic acids, mostly pyruvate and citrate, into the growth medium (Ziganshin et al. 2010b), these metabolites would probably provide the cells with some protection against oxidative damage. Pyruvic acid, like other α-keto acids, acts as a hydrogen peroxide (H_2_O_2_) scavenger and is able to react with peroxynitrite and peroxynitrous acid via pyruvate decarboxylation to acetate (Vásquez-Vivar et al. 1997). Furthermore, some protective properties of citric acid against superoxide anions (Van den Berg et al. 2003) and peroxynitrite (Han et al. 2005) are also known.

The results of our experiments also indicate a greater likelihood of ferrihydrite and magnetite to be reduced to ferrous iron in the presence of aerobic yeast cells and TNT than hematite or goethite.

Possible mechanisms for the reduction of the ferric (oxyhydr)oxides here could be the induction of yeast enzymes with Fe(III) reducing capacity by TNT or its transformation products as well as the participation of TNT degradation products and/or some unidentified extracellular metabolites in the abiotic reduction of Fe(III). Kerem et al. (1999) demonstrated that the brown rot fungus *Gloeophyllum trabeum* was able to degrade another recalcitrant compound, polyethylene glycol. *G. trabeum* excreted an extracellular metabolite, 2,5-dimethoxy-1,4-benzoquinone, into the medium and after its reduction to 2,5-dimethoxyhydroquinone, this compound led to the non-enzymatic reduction of dissolved Fe(III) to Fe(II). The production of electron shuttling compounds by microbes can also enhance metal reduction (Gerlach et al. 2011; Field et al. 2013). Similar mechanisms during TNT biodegradation by *Y. lipolytica* AN-L15 could potentially also occur.

Another possible reason for Fe(II) accumulation and changing ferric (oxyhydr)oxide mineralogy in the presence of dissolved O_2_ is the participation of reactive oxygen species in Fe(III) reduction (Melton et al. 2014). The reactivity of biologically produced O_2_^·–^ toward amorphous ferric (oxyhydr)oxides leading to Fe(II) formation was previously investigated (Kustka et al. 2005; Fujii et al. 2006). It is known that O_2_^·–^/HO_2_^·^ can reduce dissolved ferric iron at a range of pH values (Rush and Bielski 1985), and organically associated Fe(III) has also been reported to be reduced by O_2_^·–^/HO_2_^·^ (Rose and Waite 2005). In addition, a partial re-oxidation of the released Fe(II) to Fe(III) might occur by either molecular oxygen (Morgan and Lahav 2007), nitric oxide or nitrogen dioxide (Klueglein and Kappler 2013) generated during TNT biodegradation. We also cannot exclude that a fraction of the produced Fe(II) reacted with O_2_^·–^/HO_2_^·^ with the subsequent formation of Fe(III) and H_2_O_2_. However, the rate of ferric ion reduction by O_2_^·–^ is usually faster than that of ferrous iron oxidation by O_2_^·–^ (Rush and Bielski 1985). In addition, O_2_^·–^ might have reacted with NOM to form H_2_O_2_ in our systems (Fischer and Warneck 1996). Yet another possible mechanism of partial Fe(II) oxidation is related to the Fenton chemistry that involves H_2_O_2_ produced either via reaction of Fe(II) with O_2_^·–^ (Rush and Bielski 1985), via reaction with NOM (Fischer and Warneck 1996), or via superoxide, hydroperoxyl and superoxide/hydroperoxyl disproportionation reactions (Rose and Waite 2005). Fe(III) can be also reduced back to Fe(II) by H_2_O_2_ with the generation of a hydroperoxyl radical. Additionally, O_2_^·–^ could have also interacted with H_2_O_2_ to produce hydroxyl radicals (Haber and Weiss 1934). Due to the high reactivity of the different radical and peroxide species, a large number of reactions might have occurred in these systems and it is quite possible that reactions other than the described ones occurred in the experimental systems. Table 1 lists a number of possible reactions and products that might occur during TNT transformation in the presence of *Y. lipolytica* AN-L15 and Fe(III) minerals. Due to the complexity of radical-based reactions, however, the list of reactions is very likely incomplete.

**Table 1:** **Possible reactions that can take place during TNT transformation in the presence of** ***Y. lipolytica*** **AN-L15 and Fe(III) minerals**

**Biological reactions**					
TNT	**→**	3-H^−^-TNT isomers; 3,5-2H^−^-TNT; 3,5-2H^−^-TNT · H^+^ isomers; 1-H^−^-TNT			
TNT	**→**	2-HADNT; 4-HADNT; 2-ADNT; 4-ADNT			
3-H^−^-TNT	**→**	2,4-DNT + NO_2_^−^			
3,5-2H^−^-TNT · H^+^	**→**	NO_2_^−^ + ?			
1-H^−^-TNT	**→**	NO_2_^−^ + ?			
NO_2_^−^	**→**	NO_3_^−^			
**Abiotic reactions**					
3-H^−^-TNT	**⇄**	3-H^−^-TNT isomers	HO_2_^·^ + HO_2_^·^	**→**	O_2_ + H_2_O_2_
3-H^−^-TNT	**⇄**	3,5-2H^−^-TNT	HO_2_^·^ + O_2_^·–^	**→**	O_2_ + HOO^−^
3,5-2H^−^-TNT	**→**	3,5-2H^−^-TNT · H^+^ isomers	O_2_^·–^ + O_2_^·–^ + 2H^+^	**→**	O_2_ + H_2_O_2_
3-H^−^-TNT	**→**	TNT	O_2_^·–^ + NOM	**→**	H_2_O_2_ + ?
NO_2_^−^ + H^+^	**⇆**	HNO_2_	O_2_^·–^ + H_2_O_2_	**→**	HO^.^ + OH^−^ + O_2_
3HNO_2_	**→**	HNO_3_ + 2NO + H_2_O	Fe(III) + O_2_^·–^	**→**	Fe(II) + O_2_
2HNO_2_	**→**	NO_2_ + NO + H_2_O	Fe(III) + H_2_O_2_	**→**	Fe(II) + HO_2_^·^ + H^+^
2NO + O_2_	**→**	2NO_2_	Fe(II) + HO_2_^·^ + H^+^	**→**	Fe(III) + H_2_O_2_
NO_2_ + NOM^*^	**→**	?	Fe(II) + O_2_^·–^ + 2H^+^	**→**	Fe(III) + H_2_O_2_
O_2_^·–^ + NO	**→**	ONOO^−^	Fe(II) + H_2_O_2_	**→**	Fe(III) + HO^.^ + OH^−^
ONOO^−^ + H^+^	**=**	ONOOH	Fe(II) + O_2_	**→**	Fe(III) + O_2_^·–^
ONOOH	**⇆**	NO_3_^−^ + H^+^	2Fe(II) + NO_2_ + 2H^+^	**→**	2Fe(III) + NO + H_2_O
ONOO^−^ + CO_2_	**→**	NO_2_ + CO_3_^·–^	Fe(II) + NO + H^+^	**→**	Fe(III) + HNO
O_2_^·–^ + H^+^	**⇆**	HO_2_^·^	2HNO	**→**	N_2_O + H_2_O

^*^Natural organic matter.

Mössbauer spectroscopy revealed only relatively minor differences between ferrihydrite, goethite and magnetite minerals incubated with and without TNT; however, hematite exhibited no differences at all. No secondary Fe(II) mineral phases were detected despite the release of a significant proportion of aqueous Fe(II) as detected by the ferrozine assay. These results suggest that all of the Fe(II) which was released in the presence of only cells, or cells + TNT remained in solution and was not re-precipitated as Fe(II) minerals. This is likely due to the low pH of the growth medium which dropped to pH 3 over the course of the incubation. Incubation of the magnetite sample in the presence of *Y. lipolytica* AN-L15 and TNT resulted in formation of a minor mineral phase which is consistent with the Mössbauer spectrum of a poorly crystalline Fe(III) mineral phase; however, it is not clear what the mineral identity is. A decrease in the relative area of the paramagnetic component in the goethite sample was detected during incubation in the presence of *Y. lipolytica* AN-L15 and TNT. Analyses performed on both ferrihydrite samples showed that a significant fraction of the mineral in both of them is still not fully magnetically ordered even at 5 K. This would imply the samples have a very low blocking temperature, (i.e. temperature below which a mineral is magnetically ordered) likely much lower than the value which is typically expected for ferrihydrite (Murad 1996). Decreases in the blocking temperature can be caused by poor crystallinity (e.g. due to decreasing particle size) or by association with organic complexes. Mikutta et al. (2008) observed a decrease in blocking temperature from 68 K for pure ferrihydrite to 50 K for ferrihydrite synthesized in the presence of polysaccharides. Thus, it is possible that our samples were not fully magnetically ordered because they were associated with organic material; however, this effect is usually only observed when ferrihydrite is synthesized in the presence of organic matter. The decrease in the relative area of the paramagnetic background for samples containing both goethite and ferrihydrite in the presence of cells and TNT could be primarily attributed to an increase in particle crystallinity. However, this somewhat contradicts the idea that Fe is being released into solution (as Fe(II)) which would require dissolution of the mineral phase, thus leading to a decrease in the particle crystallinity. An alternative explanation could be due to a shift in the particle size distribution which may be present in the goethite and ferrihydrite minerals with a mixture of some large crystalline phases and some smaller (i.e. more poorly crystalline) phases. The smaller particles would have a larger surface area to volume ratio, could thus be more accessible to the yeast cells, and therefore more likely to be the phases which are dissolved, leading to the release of Fe which was detected as Fe(II) in solution by ferrozine. ^57^Fe Mössbauer only accounts for the solid mineral fraction; therefore as the more poorly crystalline phases are dissolved, the relative amount of more crystalline phase increases which could be interpreted as an increase in crystallinity, as seen in our data.

Our results clearly show the complexity of TNT transformation by *Y. lipolytica* AN-L15 in the presence of ferric (oxyhydr)oxides. In addition to biotic processes mediated by the yeast strain *Y. lipolytica* AN-L15, oxygen and nitrogen reactive species generated during TNT transformation process also participated in the abiotic transformation of iron and TNT. Since Fe(II)- and Fe(III)-bearing minerals are found at many TNT-contaminated field areas, the received results are significant for further understanding of TNT degradation and detoxification in the environment.

## Additional file

Additional file 1:
**Table S1.** Change of medium pH during aerobic growth of *Y. lipolytica* AN-L15 and maximum amounts of some metabolites (μM) detected over the course of TNT biotransformation. **Table S2.** Mössbauer spectroscopic analysis of various ferric (oxyhydr)oxides after their aerobic incubation with *Y. lipolytica* AN-L15 cells in the absence or presence of TNT. **Figure S1.** Formation of dissolved Fe(II) during aerobic growth of *Y. lipolytica* AN-L15 in the presence of different ferric (oxyhydr)oxides (0.15 g L^–1^ Fe Error bars represent the standard deviation of triplicate experiments.
