# Supplementary material for: Fe(III) mineral reduction followed by partial dissolution and reactive oxygen species generation during 2,4,6-trinitrotoluene transformation by the aerobic yeast Yarrowia lipolytica
Source: AMB Express. 2015 Feb 1;5:8. doi: 10.1186/s13568-014-0094-z (PMC4314830; doi:10.1186/s13568-014-0094-z)
Supplement: Additional file 1: — Table S1. Change of medium pH during aerobic growth of Y. lipolytica AN-L15 and maximum amounts of some metabolites (μM) detected over the course of TNT biotransformation. Table S2. Mössbauer spectroscopic analysis of various ferric (oxyhydr)oxides after their aerobic incubation with Y. lipolytica AN-L15 cells in the absence or presence of TNT. Figure S1. Formation of dissolved Fe(II) during aerobic growth of Y. lipolytica AN-L15 in the presence of different ferric (oxyhydr)oxides (0.15 g L–1 Fe Error bars represent the standard deviation of triplicate experiments. [file 13568_2014_94_MOESM1_ESM.pdf]

Electronic supplementary material

Fe(III) mineral reduction followed by partial dissolution and reactive oxygen species generation during 2,4,6-trinitrotoluene transformation by the aerobic yeast *Yarrowia lipolytica*

Ayrat M. Ziganshin · Elvira E. Ziganshina · James Byrne · Robin Gerlach · Ellen Struve · Timur Biktagirov · Alexander Rodionov · Andreas Kappler

A. M. Ziganshin (✉) · E. E. Ziganshina

Department of Microbiology, Kazan (Volga Region) Federal University, ul. Kremlyovskaya 18, Kazan 420008, The Republic of Tatarstan, Russia

e-mail: a.ziganshin06@fulbrightmail.org

J. Byrne · E. Struve · A. Kappler (✉)

Geomicrobiology, Center for Applied Geosciences, University of Tübingen, Tübingen D-72076, Germany

e-mail: andreas.kappler@uni-tuebingen.de

R. Gerlach

Department of Chemical and Biological Engineering, Montana State University, Bozeman, Montana 59717, USA

T. Biktagirov · A. Rodionov

Institute of Physics, Kazan (Volga Region) Federal University, Kazan 420008, The Republic of Tatarstan, Russia

Additional file 1: Table S1 Change of medium pH during aerobic growth of *Y. lipolytica* AN-L15 and maximum amounts of some metabolites (μM) detected over the course of TNT biotransformation

Sample	pH	2,4-DNT (μM)	2-HADNT (μM)	4-HADNT (μM)	ADNTs (μM)
Y. lipolytica + TNT	2.65±0.05	72±7	38±4	94±6	2±3
Y. lipolytica + goethite + TNT	2.30±0.07	37±5	48±5	91±7	8±3
Y. lipolytica + hematite + TNT	2.34±0.04	46±4	39±5	87±7	8±4
Y. lipolytica + magnetite + TNT	2.41±0.06	31±5	45±4	85±6	9±4
Y. lipolytica + ferrihydrite + TNT	2.79±0.12	15±3	44±6	95±8	12±3

Data for nitro group elimination product (2,4-DNT) and nitro group reduction metabolites (HADNTs and ANDTs) are only shown. Data for Meisenheimer hydride complexes are not presented since they were effectively biodegraded after formation from TNT. However, their initial concentrations (around 70%) were much higher than those of the TNT nitro group reduction products (around 30%). Data for pH and TNT metabolites presented in the table were recorded after 5 days of incubation.

Additional file 1: Table S2 Mössbauer spectroscopic analysis of various ferric (oxyhydr)oxides after their aerobic incubation with *Y. lipolytica* AN-L15 cells in the absence or presence of TNT

Sample	Temp. K	Phase	<CS> (mm/s)[a]	<|ΔE$_Q$|> (mm/s)[b]	<ε> (mm/s)[c]	<|H|> (T)[d]	Pop. %	±	$(\chi^2)^e$
Hematite	295	S	0.38	–	–0.11	51.4	100.0	0.0	1.62
Hematite + TNT	295	S	0.37	–	–0.11	51.4	100.0	0.0	0.86
Magnetite	295	$^{Oct}Fe^{2.5+}$	0.69	–	0.01	45.5	63.9	0.7	1.32
		$^{Tet}Fe^{3+}$	0.26	–	–0.02	49.0	36.1	0.7	
Magnetite + TNT	295	D	0.28	0.35	–	–	1.3	0.5	0.77
		$^{Oct}Fe^{2.5+}$	0.68	–	0.01	46.1	52.7	1.1	
		$^{Tet}Fe^{3+}$	0.28	–	0.00	49.3	46.0	1.1	
Goethite	295	D[f]	0.21	7.50	–	–	39.5	3.6	0.83
		S[g]	0.37	–	–0.14	33.9	60.5	3.6	
Goethite + TNT	295	D	0.38	6.77	–	–	24.8	6.0	1.05
		S	0.43	–	–0.17	33.1	75.2	6.0	
Ferrihydrite	295	D	0.38	0.68	–	–	100.0	0.0	0.60
	77	D	0.49	0.69	–	–	100.0	0.0	0.67
	5	S1	0.59	–	–0.08	47.8	16.6	3.3	0.72
		S2	0.38	–	0.01	46.1	28.4	4.0	
		S3	0.41	–	–0.11	24.1	55.0	4.2	
Ferrihydrite + TNT	295	D	0.38	0.70	–	–	100.0	0.0	0.53
	77	D	0.49	0.72	–	–	100.0	0.0	0.77
	5	S1	0.53	–	–0.09	48.1	20.0	2.9	0.81
		S2	0.40	–	0.02	45.9	30.1	3.4	
		S3	0.48	–	–0.10	27.4	49.9	3.3	

[a] Center shift; [b] Mean Quadrupole splitting; [c] Mean quadrupole shift; [d] Mean magnetic hyperfine field; [e] Fitting error; [f] Doublet; [g] Sextet.

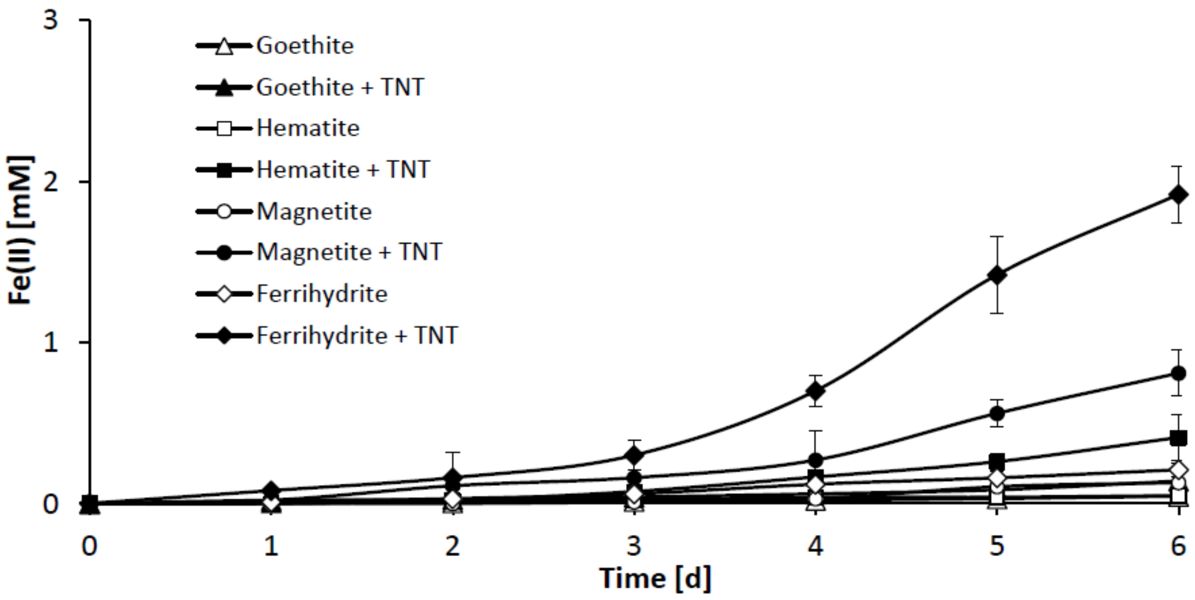

Additional file 1: Figure S1 Formation of dissolved Fe(II) during aerobic growth of *Y. lipolytica* AN-L15 in the presence of different ferric (oxyhydr)oxides (0.15 g L^{-1} Fe). Error bars represent the standard deviation of triplicate experiments.
